# Stage-specific survival has improved for young breast cancer patients since 2000: but not equally

**DOI:** 10.1007/s10549-020-05698-z

**Published:** 2020-06-03

**Authors:** Cassia Bree Trewin, Anna Louise Viktoria Johansson, Kirsti Vik Hjerkind, Bjørn Heine Strand, Cecilie Essholt Kiserud, Giske Ursin

**Affiliations:** 1 Norwegian National Advisory Unit on Women’s Health, Oslo University Hospital, Rikshospitalet, P.O. Box 4950, Nydalen, 0424 Oslo, Norway; 2 Department of Registration, Cancer Registry of Norway, P.O. Box 5313, Majorstuen, 0304 Oslo, Norway; 3 Faculty of Medicine, University of Oslo, P.O. Box 1078, Blindern, 0316 Oslo, Norway; 4 Department of Medical Epidemiology and Biostatistics, Karolinska Institutet, 171 77 Stockholm, Sweden; 5 Cancer Registry of Norway, P.O. Box 5313, Majorstuen, 0304 Oslo, Norway; 6 Department of Chronic Diseases and Ageing, Norwegian Institute of Public Health, P.O. Box 222, Skøyen, 0213 Oslo, Norway; 7 Department of Community Medicine, Institute of Health and Society, University of Oslo, P.O. Box 1078, Blindern, 0316 Oslo, Norway; 8 Norwegian National Advisory Unit on Aging and Health, Vestfold Hospital Trust, P.O. Box 2168, 3103 Tønsberg, Norway; 9 National Resource Center for Late Effects After Cancer Treatment, Oslo University Hospital, Radiumhospitalet, P.O. Box 4953, Nydalen, 0424 Oslo, Norway; 10 Department of Nutrition, Institute of Basic Medical Sciences, University of Oslo, P.O. Box 1078, Blindern, 0316 Oslo, Norway; 11 Department of Preventative Medicine, University of Southern California, 2001 North Soto Street, Los Angeles, CA 90033 USA

**Keywords:** Breast neoplasms, Stage at diagnosis, Socioeconomic factors, Relative survival, Excess mortality

## Abstract

**Purpose:**

The stage-specific survival of young breast cancer patients has improved, likely due to diagnostic and treatment advances. We addressed whether survival improvements have reached all socioeconomic groups in a country with universal health care and national treatment guidelines.

**Methods:**

Using Norwegian registry data, we assessed stage-specific breast cancer survival by education and income level of 7501 patients (2317 localized, 4457 regional, 233 distant and 494 unknown stage) aged 30–48 years at diagnosis during 2000–2015. Using flexible parametric models and national life tables, we compared excess mortality up to 12 years from diagnosis and 5-year relative survival trends, by education and income as measures of socioeconomic status (SES).

**Results:**

Throughout 2000–2015, regional and distant stage 5-year relative survival improved steadily for patients with high education and high income (high SES), but not for patients with low education and low income (low SES). Regional stage 5-year relative survival improved from 85 to 94% for high SES patients (9% change; 95% confidence interval: 6, 13%), but remained at 84% for low SES patients (0% change; − 12, 12%). Distant stage 5-year relative survival improved from 22 to 58% for high SES patients (36% change; 24, 49%), but remained at 11% for low SES patients (0% change; − 19, 19%).

**Conclusions:**

Regional and distant stage breast cancer survival has improved markedly for high SES patients, but there has been little survival gain for low SES patients. Socioeconomic status matters for the stage-specific survival of young breast cancer patients, even with universal health care.

**Electronic supplementary material:**

The online version of this article (10.1007/s10549-020-05698-z) contains supplementary material, which is available to authorized users.

## Introduction

Stage-specific and overall survival of breast cancer patients has improved over time [1, 2], probably due to advances in diagnostics and treatment. More precise diagnosis of tumor type and stage has enabled treatment to become more tailored to the patient [3–6]. New treatments have also improved the survival of patients with certain tumor subtypes, for example Herceptin has improved the survival of HER2 positive patients [7, 8]. Although breast cancer survival has been improving, there is concern that patients with low socioeconomic status (SES) have not gained as much from recent advancements [9–14].

Like several other countries, Norway has a universal tax funded health care system with the aim to minimize socioeconomic differences in access to diagnostic and treatment care. A nationwide screening program, gradually introduced during 1996–2004, has also ensured universal access to early detection of breast cancer for women aged 50–69 years in Norway. However, younger women may have different diagnostic and care seeking behavior than screen-aged women. Young women fare worse than screen-aged women in terms of breast cancer survival, even after adjustment for tumor characteristics [15–17].

Thus, we were interested to know whether access to universal health care has been sufficient to ensure that breast cancer survival has improved for young women from all socioeconomic backgrounds. Studies of socioeconomic inequalities in survival of young patients are lacking. Only a few studies have assessed socioeconomic inequalities in stage-specific survival [18–21], and none have assessed trends over time.

We took advantage of high-quality Norwegian registry data with individually linked education and income information, to compare trends over time in the stage-specific survival of young women diagnosed before entry to the Breast Cancer Screening Program. We aimed to determine whether survival improvements have reached all socioeconomic groups in a country with universal health care and national treatment guidelines.

## Materials and methods

### Study design and materials

Using a cohort study design, we assessed the relative survival of all women in Norway diagnosed with invasive breast cancer between Jan 2000 and Dec 2015 at age 30 to 48 years. This age range ensured most patients had completed their education and started earning income, but not yet been invited to mammography screening, before diagnosis. The target screening age in Norway is 50–69 years, although some counties start at 49 years. Breast cancer patients were identified via the nationwide Cancer Registry of Norway, which has had mandatory reporting of new cancer cases since 1953 and is 99% complete [22]. Demographic and socioeconomic characteristics of patients were individually linked from the Central Population Registry, National Education database and Register for Personal Tax Payers.

### Study population and follow-up

We identified 8574 potentially eligible women diagnosed with a primary invasive breast cancer (International Classification of Diseases-10 code C50). Of these, 703 (8.2%) patients were ineligible due to a prior invasive cancer diagnosis, 78 (0.9%) had non-epithelial tumors, one had a tumor that was not morphologically verified and five were registered as emigrating before their diagnosis date. Among 7787 remaining eligible women, we excluded 286 (3.7%) women (248 immigrants and 38 Norwegian) due to an unknown education or income level, leaving a final study population of 7501 breast cancer patients. Follow-up for survival started on the 15th of the month of breast cancer diagnosis and ended upon first emigration from Norway, death, after 12 years follow-up, or 31 December 2017, whichever came first.

### Education level

We categorized patients by their most recently recorded education level before diagnosis: compulsory (lower secondary school, ≤ 10 years), secondary (upper secondary school or vocational education, 11–13 years) or tertiary (university or vocational education, ≥ 14 years). Our data included education level per 1 October 1999, 2000, 2005, 2010 and 2015. Norwegian educational institutions have mandatory reporting to the National Education Database. In our cohort, education level was 99.7% complete for Norwegian-born patients but was missing for 17.3% of eligible immigrants (2.0% of all eligible patients), most likely because these immigrants had not completed any education in Norway [23].

### Income quintile

We divided patients into quintiles of average personal income during the five-year period before breast cancer diagnosis. We categorized patients by income before diagnosis since income is likely to fall after diagnosis [24]. We categorized income quintile (Q) as low (Q1), middle (Q2-Q4) or high (Q5). Our data included average annual income during 1995–1999, 2000–2004 and 2005–2009. We therefore divided patients diagnosed in 2000–2004 into quintiles of average income during 1995–1999, patients diagnosed in 2005–2009 into quintiles of average income during 2000–2004, and patients diagnosed in 2010–2015 into quintiles of average income during 2005–2009. Past income was 99.8% complete for Norwegian-born patients but was missing for 17.8% of eligible immigrants (2.1% of all eligible patients), probably because these immigrants did not reside in Norway during the period before diagnosis when income was recorded.

### Socioeconomic status

We were interested in the effect of having both low education and low income, so formed a combined SES categorization of education and income level, where we separated the lowest education and income levels from higher levels. We divided patients into four SES groups: low/low (compulsory education/Q1 income), low/high (compulsory education/Q2–Q5 income), high/low (secondary or tertiary education/Q1 income) and high/high (secondary or tertiary education/Q2–Q5 income).

### Covariates

We categorized immigration history as immigrant if patients were foreign-born with foreign-born parents, or Norwegian if otherwise. For patients diagnosed in 2005–2015, we had information on tumor grade (low = 1, medium = 2, high = 3–4) and status (positive or negative) of the estrogen receptor (ER), progesterone receptor (PR) and HER2. Criteria for determining ER, PR and HER2 status by the Cancer Registry of Norway are described elsewhere [15]. We combined information on ER, PR, HER2 and grade to classify clinical subtype as: luminal A-like (ER and/or PR positive, HER2 negative, low grade), luminal B-like/HER2− (ER and/or PR positive, HER2 negative, medium/high grade), luminal B-like/HER2+ (ER and/or PR positive, HER2 positive, any grade), HER2+ (ER and PR negative, HER2 positive, any grade) or triple-negative (ER and PR negative, HER2 negative, any grade) [25]. Subtype was set to unknown if any of ER, PR, HER2 or grade were missing.

### Stage at diagnosis

We categorized tumor stage by pathological tumor size, nodal status and metastasis (TNM), supplemented with information from clinical reports of stage according to the Surveillance Epidemiology and End Results Program [1]. We categorized stage as localized (TNM stage I; tumors localized to the breast); regional (TNM stages II-III; metastasis to regional lymph nodes or to skin and/or chest wall); distant (TNM stage IV; metastasis to distant lymph nodes or other organs) or unknown (pathological and clinical reports were missing or incomplete). We combined TNM stages II and III because the coding practice for lymph node spread was updated at the Cancer Registry of Norway in 2008, leading to a migration between TNM stages II and III.

### Statistical analysis

We used Pearson’s Chi-squared tests to determine associations between socioeconomic variables and covariates (tumor stage, age group, diagnostic period, immigration history and clinical subtype). Associations between socioeconomic variables and breast cancer death were determined by relative survival methods, which estimate excess mortality rates due to breast cancer by comparing the observed all-cause mortality rates of patients to the expected all-cause mortality rates for females in the Norwegian population of the same age and calendar year. In preliminary analyses, we used life tables stratified by age, calendar year and socioeconomic variables to avoid bias [26]. The SES-stratified life tables were created from individually linked nationwide data of mortality, education and income, and smoothed using a multivariable flexible Poisson model [27]. We found, however, that relative survival estimates were similar when using national life tables, so therefore used the simpler un-stratified national life tables in all analyses.

We first estimated stage-specific socioeconomic inequalities in excess mortality pooled over the study period (2000–2015). We used flexible parametric models [28, 29] to estimate stage-specific excess mortality rate ratios, with 95% confidence intervals (CI), by education, income and SES group, while adjusting for age and year at diagnosis. Immigration history and clinical subtype were assessed, but not included in final models because neither were important confounders or mediators of the main effects of education, income or SES group. In all models, the baseline hazard spline utilized four degrees of freedom and varied by stage at diagnosis with two degrees of freedom [28, 29]. Year at diagnosis was modeled non-linearly using restricted cubic splines with two degrees of freedom [30]. Modeling with splines allowed us to capture any changes in the rate of survival gain at a certain time points, for example after implementation of a new treatment. Three-way interactions between year, stage and socioeconomic variable allowed rates of survival gain to vary by both stage and socioeconomic group.

From these flexible parametric models, we made model-based predictions of 5-year relative survival with 95% confidence intervals (CI) for patients aged 40 years at diagnosis. We first predicted stage-specific 5-year relative survival over time for each socioeconomic group, then predicted difference in 5-year relative survival between the highest and lowest socioeconomic groups in 2000 and 2015. For patients diagnosed in 2015, we also made model-based predictions of relative survival up to 12 years from diagnosis. These 2015 predictions are outside the scope of the data and hence based on model parameters, so for comparison we calculated non-parametric Pohar Perme estimates of net survival [31] for patients diagnosed during 2005–2015 (Online Resource 1 and 2). The results were similar between model-based and non-parametric estimates.

We performed our analysis using STATA version 15.1 (StataCorp LLC, College Station, TX, USA, RRID:SCR_012763) [32]. We considered a two-sided *p *value less than 0.05 as statistically significant. Ethical approval was obtained from the Regional Committee for Medical and Health Research Ethics in Norway (Ref. 2013/2376). The dataset is managed in accordance with the European General Data Protection Regulation (GDPR).

## Results

This study included 7501 patients, among whom we observed 1117 excess deaths due to breast cancer over 58418 person-years follow-up from diagnosis. There were 2317 (30.9%) patients with localized stage, 4457 (59.4%) with regional stage, 233 (3.1%) with distant stage, and 494 (6.6%) with unknown stage at diagnosis. Average follow-up per patient diagnosed with localized, regional, distant and unknown stage breast cancer was 8.3, 7.7, 3.4 and 7.8 years, respectively. High education was associated with more recent diagnosis (*p* < 0.001) and younger age at diagnosis (*p* < 0.001), while high income was associated with older age at diagnosis (*p* < 0.001) (Table 1). Neither education (*p* = 0.336) nor income (*p* = 0.376) were associated with tumor subtype.

**Table 1:** Breast cancer patient and tumor characteristics (*n* = 7501)

	Total		Education level							Income quintile						
			Compulsory		Secondary		Tertiary		Chi^2^ P	Q1 (low)		Q2–Q4		Q5 (high)		Chi^2^ P
Covariate	*n*	(%)	*n*	(%)	*n*	(%)	*n*	(%)	*χ*^2^*p*	*n*	(%)	*n*	(%)	*n*	(%)	*χ*^2^*p*
Total	7501	(100)	1475	(100)	2896	(100)	3130	(100)		1499	(100)	4500	(100)	1502	(100)	
Stage																
Localized	2317	(33.1)	430	(31.2)	885	(32.8)	1002	(34.3)	0.079	398	(28.8)	1417	(33.7)	502	(35.2)	< 0.001
Regional	4457	(63.6)	892	(64.6)	1726	(63.9)	1839	(62.9)		913	(66.1)	2656	(63.3)	888	(62.3)	
Distant	233	(3.3)	58	(4.2)	91	(3.4)	84	(2.9)		71	(5.1)	126	(3.0)	36	(2.5)	
Unknown^a^	494		95		194		205			117		301		76		
Diagnosis period																
2000–2004	2177	(29.0)	502	(34.0)	930	(32.1)	745	(23.8)	< 0.001	435	(29.0)	1306	(29.0)	436	(29.0)	1.000
2005–2009	2276	(30.3)	514	(34.8)	871	(30.1)	891	(28.5)		455	(30.4)	1365	(30.3)	456	(30.4)	
2010–2015	3048	(40.6)	459	(31.1)	1095	(37.8)	1494	(47.7)		609	(40.6)	1829	(40.6)	610	(40.6)	
Age at diagnosis																
30–34 years	469	(6.3)	70	(4.7)	165	(5.7)	234	(7.5)	< 0.001	147	(9.8)	291	(6.5)	31	(2.1)	< 0.001
35–59 years	1276	(17.0)	213	(14.4)	455	(15.7)	608	(19.4)		269	(17.9)	834	(18.5)	173	(11.5)	
40–44 years	2575	(34.3)	507	(34.4)	989	(34.2)	1079	(34.5)		499	(33.3)	1491	(33.1)	585	(38.9)	
45–48 years	3181	(42.4)	685	(46.4)	1287	(44.4)	1209	(38.6)		584	(39.0)	1884	(41.9)	713	(47.5)	
Immigration history																
Norwegian	6849	(91.3)	1311	(88.9)	2705	(93.4)	2833	(90.5)	< 0.001	1218	(81.3)	4206	(93.5)	1425	(94.9)	< 0.001
Immigrant	652	(8.7)	164	(11.1)	191	(6.6)	297	(9.5)		281	(18.7)	294	(6.5)	77	(5.1)	
Tumor subtype, 2005–2015																
Luminal A-like	2239	(49.2)	409	(50.0)	815	(48.0)	1015	(49.9)	0.336	423	(47.1)	1327	(48.8)	489	(52.6)	0.376
Luminal B-like/HER2−	775	(17.0)	132	(16.1)	294	(17.3)	349	(17.2)		154	(17.1)	468	(17.2)	153	(16.5)	
Luminal B-like/HER2 +	677	(14.9)	108	(13.2)	256	(15.1)	313	(15.4)		139	(15.5)	407	(15.0)	131	(14.1)	
HER2+	262	(5.8)	48	(5.9)	96	(5.7)	118	(5.8)		55	(6.1)	153	(5.6)	54	(5.8)	
Triple negative	596	(13.1)	121	(14.8)	237	(14.0)	238	(11.7)		128	(14.2)	366	(13.5)	102	(11.0)	
Unknown^b^	764		154		265		345			162		467		135		

^a^Missing stage: Compulsory: 6.4%, secondary: 6.7%, tertiary: 6.5%; Q1 income: 7.8%, Q2–Q4: 6.7%, Q5: 5.1%

^b^Missing subtype: Compulsory: 15.9%, secondary: 13.6%, tertiary: 14.7%; Q1 income: 15.4%, Q2–Q4: 14.8%, Q5: 12.8%

### Stage-specific excess mortality

In all socioeconomic groups, excess mortality rates were clearly highest at distant stage, but regional stage accounted for the greatest number of excess deaths, because of the high number of patients diagnosed at regional stage (Table 2). After adjustment for diagnosis age and year, excess mortality due to regional and distant stage breast cancer was significantly higher for compulsory versus tertiary educated patients, and for patients in the lowest and middle quintiles compared to the highest income quintile. There was a tendency for greater educational and income inequalities in excess mortality with more advanced stage at diagnosis.

**Table 2:** Stage-specific excess mortality of breast cancer patients up to 12 years from diagnosis, by education level and income quintile. Patients with a known stage at diagnosis (*n* = 7007)

		No. of patients	No. of deaths	Excess mortality rate per 1000 person-years	Excess mortality rate ratio^a^ (95% CI)	*p *value^b^
Stage	Education level					
Localized	Compulsory	430	33	7.6	1.67 (0.95–2.93)	0.151
	Secondary	885	56	6.2	1.05 (0.62–1.79)	
	Tertiary	1002	46	4.8	1	
Regional	Compulsory	892	216	29.2	1.57 (1.27–1.95)	< 0.001
	Secondary	1726	291	20.1	0.91 (0.74–1.12)	
	Tertiary	1839	292	20.4	1	
Distant	Compulsory	58	55	417.1	2.44 (1.66–3.59)	< 0.001
	Secondary	91	63	185.0	1.07 (0.75–1.55)	
	Tertiary	84	58	173.5	1	

Stage	Income quintile					
Localized	Q1 (low)	398	26	6.5	1.27 (0.63–2.53)	0.609
	Q2–Q4	1417	81	5.7	0.95 (0.53–1.70)	
	Q5 (high)	502	31	6.1	1	
Regional	Q1 (low)	913	182	25.4	1.68 (1.23–2.28)	0.005
	Q2–Q4	2656	488	22.7	1.41 (1.07–1.87)	
	Q5 (high)	888	130	17.3	1	
Distant	1 (low)	71	59	269.4	2.23 (1.31–3.78)	0.012
	Q2–Q4	126	96	224.7	1.78 (1.07–2.94)	
	Q5 (high)	36	21	131.0	1	

^a^Age and year adjusted rate ratios of the excess mortality of breast cancer patients, compared to the expected mortality for the Norwegian female population of the same age and calendar year. Estimated from flexible parametric models

^b^Wald test for overall significance of education/income at each stage

### Trends in stage-specific 5-year relative survival

**Fig. 1:**
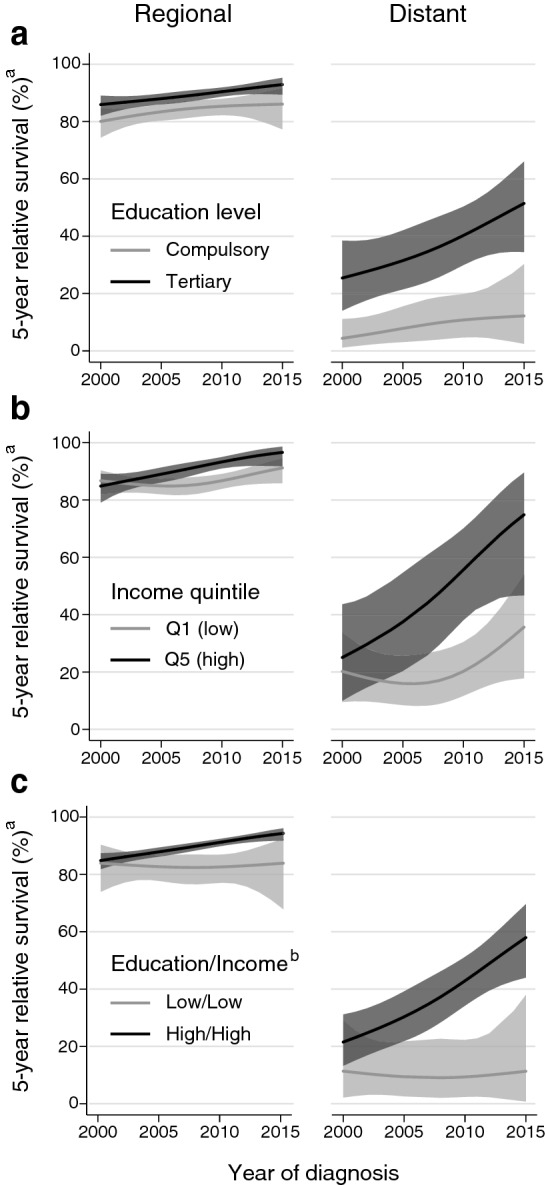
Trends in regional and distant stage 5-year relative survival, for **a** compulsory and tertiary educated patients (*n* = 2873); **b** patients in income quintiles Q1 and Q5 (*n* = 1908); and **c)** patients with compulsory education/Q1 income and secondary-tertiary education/Q2-Q5 income (*n* = 3425). ^a^Model-based predictions of relative survival, with 95% CI, for patients aged 40 years at diagnosis, compared to expected survival for the Norwegian female population. Note that predictions after 2012 are outside the scope of the data. ^b^Education/Income group: Low/Low = Compulsory/Income quintile Q1; High/High = Secondary-Tertiary/Income quintiles Q2–Q5

Over time, regional and distant stage 5-year relative survival improved the least for patients with compulsory education and for patients in the lowest income quintile (Table 3, Fig. 1a and b). Patients with both low (compulsory) education and low (Q1) income had no improvement at all over time in 5-year relative survival from localized, regional or distant stage disease (Table 4, Fig. 1c). Educational and income differences in 5-year relative survival widened particularly over time for distant stage disease. Between 2000 and 2015, the difference in distant stage relative survival widened from 21 to 39% for tertiary versus compulsory educated patients, from 5 to 39% for patients in the highest versus lowest income quintiles, and from 10 to 47% for patients with high education and high income versus low education and low income.

**Table 3:** Model-based predictions of stage-specific five-year relative survival of breast cancer patients, by education level and income quintile. Patients with a known stage at diagnosis (*n* = 7007)

		Estimated five-year relative survival^a^		Change in relative survival
		2000	2015	2000 to 2015
		% (95% CI)	% (95% CI)	% (95% CI)
Stage	Education level			
Localized	Compulsory	96 (93, 98)	97 (95, 99)	1 (0, 3)
	Secondary	97 (95, 98)	99 (98, 100)	2 (1, 3)
	Tertiary	97 (96, 98)	99 (98, 99)	1 (0, 2)
	Tertiary–Compulsory	1 (− 1, 4)	1 (− 1, 3)	
Regional	Compulsory	80 (74, 85)	86 (77, 92)	6 (− 1, 13)
	Secondary	85 (82, 88)	95 (92, 97)	10 (7, 13)
	Tertiary	86 (82, 89)	93 (89, 95)	7 (3, 11)
	Tertiary–Compulsory	6 (0, 12)	7 (− 1, 14)	
Distant	Compulsory	4 (1, 11)	12 (2, 30)	8 (− 5, 21)
	Secondary	19 (10, 29)	61 (42, 75)	42 (26, 58)
	Tertiary	26 (14, 38)	51 (35, 66)	26 (11, 41)
	Tertiary–Compulsory	21 (8, 34)	39 (18, 61)	

Stage	Income quintile			
Localized	Q1 (low)	97 (95, 99)	98 (96, 99)	1 (0, 2)
	Q2–Q4	97 (95, 98)	99 (98, 99)	2 (1, 3)
	Q5 (high)	96 (93, 98)	99 (98, 100)	3 (1, 5)
	Q5 (high)–Q1 (low)	− 1 (− 4, 1)	1 (− 1, 2)	
Regional	Q1 (low)	87 (82, 90)	91 (86, 95)	4 (0, 9)
	Q2–Q4	83 (80, 86)	93 (90, 95)	10 (7, 13)
	Q5 (high)	85 (79, 89)	97 (92, 99)	12 (7, 17)
	Q5 (high)–Q1 (low)	− 2 (− 8, 4)	5 (0, 11)	
Distant	Q1 (low)	21 (10, 34)	36 (18, 54)	15 (− 3, 34)
	Q2–Q4	14 (8, 22)	48 (33, 61)	33 (21, 46)
	Q5 (high)	25 (10, 44)	75 (47, 90)	50 (29, 71)
	Q5 (high)–Q1 (low)	5 (− 17, 26)	39 (11, 67)	

^a^Estimated relative survival of breast cancer patients five years after diagnosis, compared to expected survival for the Norwegian female population. Predicted for patients aged 40 years at diagnosis in 2000 and 2015. Note that 2015 predictions are outside the scope of the data. See Supplementary Table 1 for non-parametric relative survival estimates

**Table 4:** Model-based predictions of stage-specific five-year relative survival of breast cancer patients, by education/income group. Patients with a known stage at diagnosis (*n* = 7007)

Stage		Estimated five-year relative survival^b^		Change in relative survival
		2000	2015	2000 to 2015
	Education/Income^a^	% (95% CI)	% (95% CI)	% (95% CI)
Localized	Low/Low	98 (94, 100)	98 (93, 100)	0 (− 1, 1)
	Low/High	95 (91, 97)	97 (94, 99)	3 (0, 5)
	High/Low	97 (94, 98)	98 (96, 99)	1 (0, 3)
	High/High	97 (96, 98)	99 (98, 99)	2 (1, 3)
	High/High–Low/Low	− 1 (− 3, 1)	1 (− 2, 3)	
Regional	Low/Low	84 (74, 90)	84 (68, 92)	0 (− 12, 12)
	Low/High	79 (71, 84)	89 (77, 95)	10 (1, 19)
	High/Low	88 (82, 92)	93 (87, 96)	5 (− 1, 10)
	High/High	85 (82, 87)	94 (92, 96)	9 (6, 13)
	High/High–Low/Low	1 (− 7, 9)	10 (− 1, 22)	
Distant	Low/Low	11 (2, 29)	11 (1, 38)	0 (− 19, 19)
	Low/High	2 (0, 8)	13 (1, 40)	12 (− 9, 32)
	High/Low	26 (10, 45)	47 (22, 68)	20 (− 3, 44)
	High/High	22 (13, 31)	58 (44, 70)	36 (24, 49)
	High/High–Low/Low	10 (− 6, 27)	47 (23, 70)	

^a^Education/Income group: Low/Low: Compulsory/Income quintile Q1; Low/High: Compulsory/ Income quintiles Q2–Q5; High/Low: Secondary–Tertiary/Q1; High/High: Secondary–Tertiary/Q2–Q5

^b^Estimated relative survival of breast cancer patients five years after diagnosis, compared to expected survival for the Norwegian female population. Predicted for patients aged 40 years at diagnosis in 2000 and 2015. Note that 2015 predictions are outside the scope of the data. See Supplementary Table 1 for non-parametric relative survival estimates

**Fig. 2:**
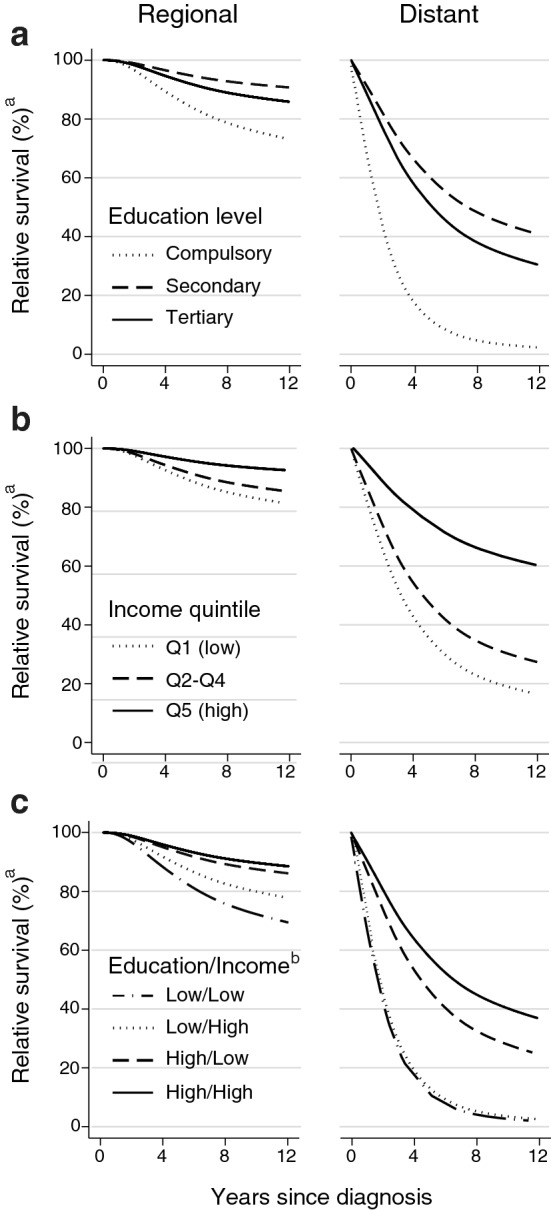
Model-based predictions of regional and distant stage relative survival for breast cancer patients aged 40 years at diagnosis in 2015, by **a** education level; **b** income quintile; and **c** education/income group (*n* = 7007). ^a^Relative survival of patients compared to the expected survival for the Norwegian female population. Note that predictions beyond two years after diagnosis are outside the scope of the data, see Online Resource 1 for non-parametric relative survival curves. ^b^Education/Income group: Low/Low: Compulsory/Income quintile Q1; Low/High: Compulsory/ Income quintiles Q2–Q5; High/Low: Secondary–Tertiary/Q1; High/High: Secondary–Tertiary/Q2–Q5

By 2015, model-based predictions of regional and distant stage relative survival were clearly better for tertiary and secondary compared to compulsory educated patients (Fig. 2a) and for patients in the highest income quintile versus the middle and lowest income quintiles (Fig. 2b). When education and income were examined in combination, we found both socioeconomic factors influenced regional stage relative survival, but education seemed a stronger predictor than income of distant stage relative survival (Fig. 2c). In 2015, the 5-year relative survival predictions for patients with low/low, low/high, high/low and high/high education/income level were, respectively: 98%, 97%, 98% and 99% for localized disease; 84%, 89%, 93% and 94% for regional stage disease; and 11%, 13%, 47% and 58% for distant stage disease (Table 4).

## Discussion

In Norway, a country with universal health care and national treatment guidelines, regional and distant stage survival improved more rapidly over time for young breast cancer patients with high SES compared to those with low SES. This widening survival gap over time between high and low SES patients was most pronounced for patients with distant spread at diagnosis. Survival from localized breast cancer was high for all socioeconomic groups throughout the study period, 2000–2015.

The reasons why low SES women lag behind high SES women in terms of survival gain are likely multifactorial. Potential reasons may include lifestyle, comorbidity, participation in clinical trials, differential access to new treatments, the opportunity or ability of patients to make informed treatment choices, motivation to adhere to treatment, or quality of care and follow-up provided by physicians. We and others [33, 34] have found no association between SES and tumor type, indicating that biological differences are unlikely to explain the association between SES and stage-specific survival.

Delayed access to new treatments may have delayed survival improvements for low SES patients [35]. There is evidence that differential treatment contributes to socioeconomic inequalities in survival, also in countries with universal health care [36, 37]. Despite universal health care and national treatment guidelines in Norway, high SES cancer patients have been reported to receive more hospital-based medical services [38] and more palliative radiotherapy [39], and high SES lung cancer patients have received more surgery and radiotherapy than low SES patients [40]. Similar surgical and radiotherapy differences have also been reported for breast cancer patients in the United Kingdom, where health care is also universal [41]. In Sweden, high SES patients were more likely to receive breast conserving surgery over mastectomy [42]. A recent study in the United Kingdom suggests that differential treatment contributes more to breast cancer survival inequalities than previously thought [36].

Scandinavian studies have found that comorbidity only plays a minor role in socioeconomic inequalities in breast cancer survival [43, 44]. On the other hand, lifestyle-related factors, such as overweight, smoking and alcohol, may partly explain poorer survival of low SES patients [44]. Unhealthy behavior has also been hypothesized to reduce the ability of low SES patients to respond to treatment [37]. If true, then a less healthy lifestyle may have potentially hindered low SES patients from benefitting from new treatments to the same extent as high SES patients.

For distant stage patients, education mattered more than income for survival. In a country with universal health care, survival inequalities may therefore not be about high SES patients affording better treatment, but about making better treatment choices. Particularly in the modern world, where treatment is becoming more personalized and complex, and the pros and cons must be continually weighed up by the patient and clinician. More educated patients may be more able to acquire knowledge about their diagnosis and take an active role in their treatment choices. A review of factors influencing socioeconomic inequalities in cancer survival [37] found that more affluent cancer patients communicated better with health care professionals than socioeconomically deprived patients. Affluent patients were also more likely to receive information from hospital specialists, had better psychological health and increased social support, which led to appropriate treatment being sought. Physicians may therefore need to pay more attention to socioeconomically deprived patients to ensure they receive equal access and standard of care [45].

Our findings of better regional and distant stage survival for high compared to low SES patients were in line with earlier studies of stage-specific survival from the USA [20], Netherlands [18] and Sweden [21]. However, we found no significant survival differences for localized disease, in contrast to earlier studies, possibly because we focused on young patients, whereas earlier studies included patients of all ages [18, 21] or only those over 55 years [20]. Nevertheless, our observation of better survival for high SES patients at regional and distant stage, where most deaths occurred, suggests that equal access to health care was not sufficient to offset any effect of SES on patient survival after diagnosis.

One important question is where there would be most to gain, by reducing SES differences in regional or distant stage survival? We found greater survival inequalities for distant stage patients than for regional stage patients, in line with earlier studies [18, 21]. However, twenty times more patients were diagnosed at regional stage compared to distant stage. The greatest number of deaths therefore occurred among patients with regional stage disease. Efforts to improve the regional stage survival of low SES patients would therefore be most effective for reducing breast cancer mortality in the population.

Our study had some limitations. We lacked information on lifestyle and treatment, so were not able to determine whether these factors explained our findings. Also, patient income was only available as five-year averages, so may not have reflected actual income at the time of diagnosis. However, income over five years may be reasonably correlated with accumulated disposable wealth at the time of diagnosis. Another potential study limitation was that some subgroups were small, particularly the number of distant stage patients. We nevertheless believe that our models for distant stage gave a good estimation of the true survival trends because we observed quite similar trends for regional stage, where patient numbers were much higher than for distant stage.

A major strength of our study was the population-wide registry data of high quality and completeness [22]. We had individually linked information on socioeconomic background and virtually complete follow-up of breast cancer patients for migration and death. Our life tables were constructed from individually linked demographic, migration and mortality data for the entire female Norwegian population. From an international perspective, Norwegian Cancer Registry data have high quality, with a very high proportion of morphologically verified cancers and a very small proportion identified through death certificate only [46], demonstrating high validity of our Cancer Registry data [47]. Further, a low proportion of our patient population had unknown stage at diagnosis, and the survival of these patients did not vary by SES. Thus, selection bias due to missing stage information was unlikely to explain our findings.

## Conclusions

Despite Norway having universal health care and national treatment guidelines, we found that young breast cancer patients with low SES lag behind, with less improved regional and distant stage survival over time. Why socioeconomic status still matters for survival, even with equal health care access, is likely multifactorial and deserves more attention. Given the number of patients with regional stage disease, improving the survival of low SES patients with regional stage breast cancer would be most effective for reducing breast cancer mortality in the population.

## Electronic supplementary material

Below is the link to the electronic supplementary material.

Supplementary file1 (PDF 78 kb)

Supplementary file2 (PDF 123 kb)

## Data Availability

The data that support the findings of this study are available from the Cancer Registry of Norway but restrictions apply to the availability of these data, which were used under license for the current study, and so are not publicly available. Data are however available from the authors onsite at the Cancer Registry of Norway upon reasonable request and with permission of the Regional Committee for Medical and Health Research Ethics in Norway.

## Abbreviations

SES: Socioeconomic status
HER2: Human epidermal growth factor receptor 2
ER: Estrogen receptor
PR: Progesterone receptor
Q: Quintile
TNM: Tumor size, nodal status and metastasis
CI: Confidence interval
