# Supplementary material for: Stage-specific survival has improved for young breast cancer patients since 2000: but not equally
Source: Breast Cancer Res Treat. 2020 Jun 3;182(2):477–89. doi: 10.1007/s10549-020-05698-z (PMC7297859; doi:10.1007/s10549-020-05698-z)
Supplement: Supplementary file 1 — Supplementary file1 (PDF 78 kb) [file 10549_2020_5698_MOESM1_ESM.pdf]

Online Resource 1

Non-parametric Pohar Perme estimates of stage-specific relative survival up to 12 years from diagnosis, by **a)** education level, **b)** income quintile and **c)** combined education/income group. Patients diagnosed at 30-48 years during 2005-2015 (n = 4,985).

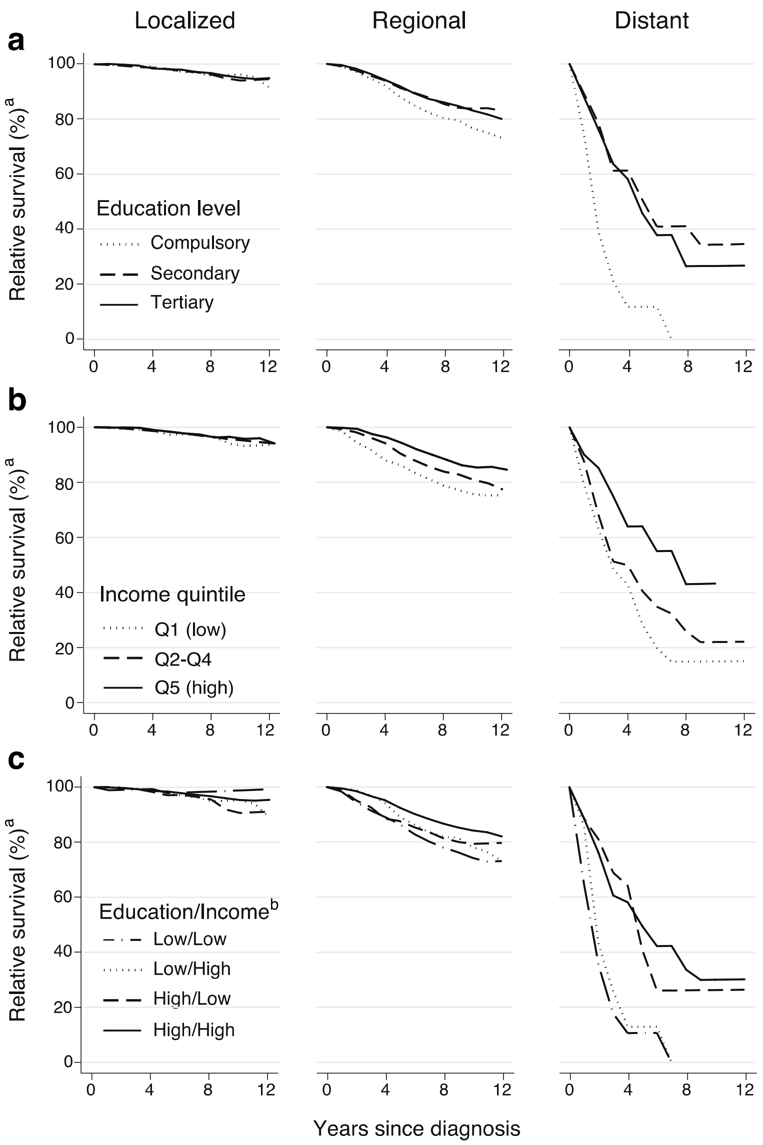

[a]Pohar Perme estimates of relative survival of breast cancer patients, compared to the expected survival of the Norwegian female population of the same age and calendar year as the patients.

[b]Education/Income group: Low/Low: Compulsory/Income quintile Q1; Low/High: Compulsory/ Income quintiles Q2-Q5; High/Low: Secondary-Tertiary/Q1; High/High: Secondary-Tertiary/Q2-Q5.

Journal: Breast Cancer Research and Treatment

Title: Stage-specific survival has improved for young breast cancer patients since 2000: but not equally

Authors: Cassia Bree Trewin, Anna Louise Viktoria Johansson, Kirsti Vik Hjerkind, Bjørn Heine Strand, Cecilie Essholt Kiserud, Giske Ursin.

Corresponding author: Cassia Bree Trewin, Cancer Registry of Norway, cassie.trewin@kreftregisteret.no
