# Supplementary material for: Stage-specific survival has improved for young breast cancer patients since 2000: but not equally
Source: Breast Cancer Res Treat. 2020 Jun 3;182(2):477–89. doi: 10.1007/s10549-020-05698-z (PMC7297859; doi:10.1007/s10549-020-05698-z)
Supplement: Supplementary file 2 — Supplementary file2 (PDF 123 kb) [file 10549_2020_5698_MOESM2_ESM.pdf]

Online Resource 2

Non-parametric Pohar Perme estimates of stage-specific 5-year relative survival, by education level, income quintile and combined education/income group. Breast cancer patients aged 30-48 years at diagnosis during 2000-2004 or 2005-2015, with a known stage at diagnosis (N = 7007).

		Estimated five-year relative survival[a]				Change in relative survival 2000-2004 to 2005-2015
		Diagnosed during 2000-2004		Diagnosed during 2005-2015		
		%	(95% CI)	%	(95% CI)	%
Stage	Education level					
Localized	Compulsory	97	(91, 99)	98	(95, 99)	1
	Secondary	97	(94, 99)	98	(96, 99)	1
	Tertiary	99	(94, 100)	98	(97, 99)	-1
	Tertiary – Compulsory	2		0		
Regional	Compulsory	83	(78, 87)	88	(85, 87)	5
	Secondary	87	(84, 90)	91	(84, 90)	4
	Tertiary	86	(83, 89)	92	(83, 89)	6
	Tertiary – Compulsory	3		4		
Distant	Compulsory	15	(5, 30)	12	(3, 27)	-3
	Secondary	17	(7, 30)	51	(36, 64)	34
	Tertiary	17	(6, 34)	46	(32, 59)	29
	Tertiary – Compulsory	2		34		
Stage	Income quintile					
Localized	Q1 (low)	99	(90, 100)	97	(94, 99)	-2
	Q2-Q4	98	(95, 99)	98	(97, 99)	0
	Q5 (high)	96	(91, 99)	98	(95, 99)	2
	Q5 (high) – Q1 (low)	-3		1		
Regional	Q1 (low)	87	(82, 91)	87	(84, 90)	0
	Q2-Q4	85	(82, 87)	91	(89, 92)	4
	Q5 (high)	88	(83, 91)	95	(92, 96)	7
	Q5 (high) – Q1 (low)	1		8		
Distant	Q1 (low)	18	(7, 34)	29	(15, 44)	11
	Q2-Q4	11	(4, 22)	41	(29, 51)	30
	Q5 (high)	29	(9, 52)	64	(39, 81)	35
	Q5 (high) – Q1 (low)	11		35		
Stage	Education/Income[b]					
Localized	Low/Low	101	(101, 101)	98	(88, 101)	-3
	Low/High	96	(89, 98)	98	(94, 98)	2
	High/Low	98	(88, 100)	97	(92, 100)	-1
	High/High	98	(95, 99)	98	(97, 99)	0
	High/High – Low/Low	-3		0		

Regional	Low/Low	86	(77, 92)	86	(81, 91)	0
	Low/High	82	(76, 86)	89	(85, 92)	7
	High/Low	87	(81, 92)	88	(84, 91)	1
	High/High	87	(84, 89)	92	(91, 94)	5
	High/High – Low/Low	1		6		
Distant	Low/Low	34	(11, 59)	11	(2, 30)	-23
	Low/High	0		13	(1, 39)	13
	High/Low	6	(1, 23)	41	(20, 61)	35
	High/High	21	(11, 33)	50	(38, 60)	29
	High/High – Low/Low	-13		39		

[a]Pohar Perme estimates of relative survival of breast cancer patients five years after diagnosis, compared to the expected survival of the Norwegian female population of the same age and calendar year as the patients.

[b]Education/Income group: Low/Low: Compulsory/Income quintile Q1; Low/High: Compulsory/ Income quintiles Q2-Q5; High/Low: Secondary-Tertiary/Q1; High/High: Secondary-Tertiary/Q2-Q5.

Journal: Breast Cancer Research and Treatment

Title: Stage-specific survival has improved for young breast cancer patients since 2000: but not equally

Authors: Cassia Bree Trewin, Anna Louise Viktoria Johansson, Kirsti Vik Hjerkind, Bjørn Heine Strand, Cecilie Essholt Kiserud, Giske Ursin.

Corresponding author: Cassia Bree Trewin, Cancer Registry of Norway, cassie.trewin@kreftregisteret.no
